# Extremely low nucleotide diversity in the X-linked region of papaya caused by a strong selective sweep

**DOI:** 10.1186/s13059-016-1095-9

**Published:** 2016-11-28

**Authors:** Robert VanBuren, Ching Man Wai, Jisen Zhang, Jennifer Han, Jie Arro, Zhicong Lin, Zhenyang Liao, Qingyi Yu, Ming-Li Wang, Francis Zee, Richard C. Moore, Deborah Charlesworth, Ray Ming

**Affiliations:** 1 FAFU and UIUC-SIB Joint Center for Genomics and Biotechnology, Fujian Provincial Key Laboratory of Haixia Applied Plant Systems Biology Fujian Agriculture and Forestry University, Fuzhou, Fujian 350002 China; 2 Department of Plant Biology, University of Illinois at Urbana-Champaign, Urbana, IL 61801 USA; 3 Texas A&M AgriLife Research, Department of Plant Pathology & Microbiology, Texas A&M University System, Dallas, TX 75252 USA; 4 Hawaii Agriculture Research Center, Kunia, HI 96759 USA; 5 USDA-ARS, Pacific Basin Agricultural Research Center, Hilo, HI 96720 USA; 6 Department of Botany, Miami University, Oxford, OH 45056 USA; 7 Institute of Evolutionary Biology, University of Edinburgh, Edinburgh, EH9 3JT UK

## Abstract

**Background:**

The papaya Y-linked region showed clear population structure, resulting in the detection of the ancestral male population that domesticated hermaphrodite papayas were selected from. The same populations were used to study nucleotide diversity and population structure in the X-linked region.

**Results:**

Diversity is very low for all genes in the X-linked region in the wild dioecious population, with nucleotide diversity *π*_syn_ = 0.00017, tenfold lower than the autosomal region (*π*_syn_ = 0.0017) and 12-fold lower than the Y-linked region (*π*_syn_ = 0.0021). Analysis of the X-linked sequences shows an undivided population, suggesting a geographically wide diversity-reducing event, whereas two subpopulations were observed in the autosomes separating gynodioecy and dioecy and three subpopulations in the Y-linked region separating three male populations. The extremely low diversity in the papaya X-linked region was probably caused by a recent, strong selective sweep before domestication, involving either the spread of a recessive mutation in an X-linked gene that is beneficial to males or a partially dominant mutation that benefitted females or both sexes. Nucleotide diversity in the domesticated X samples is about half that in the wild Xs, probably due to the bottleneck when hermaphrodites were selected during domestication.

**Conclusions:**

The extreme low nucleotide diversity in the papaya X-linked region is much greater than observed in humans, great apes, and the neo-X chromosome of *Drosophila miranda*, which show the expected pattern of Y-linked genes < X-linked genes < autosomal genes; papaya shows an unprecedented pattern of X-linked genes < autosomal genes < Y-linked genes.

**Electronic supplementary material:**

The online version of this article (doi:10.1186/s13059-016-1095-9) contains supplementary material, which is available to authorized users.

## Background

Sex chromosomes with recombination-suppressed sex-linked regions are found in all major eukaryotic lineages and have evolved independently numerous times, including in several plant species [1]. Suppressed recombination causes sex chromosomes to evolve differently from autosomes. First, some genes are restricted to one sex (for example, Y-specific genes are never present in females). Second, the effective population size (*N*_e_) is lower for sex-linked regions than for autosomal, or pseudo-autosomal, ones (except for sequences extremely closely linked to the fully Y-linked region [2]). In a population with a 1:1 sex ratio, there are three X chromosomes and one Y chromosome, relative to four of each autosome, so that the expected X and Y *N*_e_ values are thus 3/4 and 1/4, respectively, of the autosomal *N*_e_. A lower *N*_e_ makes genetic drift more important for Y-linked and, to a lesser extent, X-linked genes than for autosomal ones. A third important difference between recombining genome regions and ones with suppressed recombination, including sex-linked regions, is that positive and purifying selection, respectively, reduce neutral diversity through selective sweeps and/or background selection, collectively called “genetic hitchhiking”; this is equivalent to a further reduction in *N*_e_ [3, 4]. These processes affect Y-linked sequences more than X-linked ones, but, in species where recombination occurs in both sexes, the X recombines less than autosomal genome regions because it recombines only in females. Overall, therefore, X-linked sequences are also expected to have lower *N*_e_ than autosomal ones.

Because of their low *N*_e_, Y and X chromosomes are predicted to have lower neutral diversity than autosomes or the pseudo-autosomal region [5]. Lower X-linked than autosomal diversity is indeed found in the fruit flies *Drosophila simulans* and *Drosophila melanogaster* [6, 7], and Y linked diversity is low in humans and other mammals, *Drosophila miranda*, and the plant *Silene latifolia* [8–11].

This study examines DNA sequence diversity in sex-linked genes in papaya (*Carica papaya*). Natural papaya populations are dioecious, with genetic sex determination and XY males and XX females. Cultivated varieties are mostly gynodioecious with XY^h^ hermaphrodites and XX female. The papaya fully sex-linked region occupies about 13% of the XY chromosome pair. A Y-linked region of 8.1 Mbp is found in hermaphrodites and males (the very similar Y^h^ and Y regions, respectively, also called HSY and MSY), and its X-linked counterpart is only 3.5 Mb [12]. The rest of the chromosome consists of two large recombining pseudo-autosomal regions (PARs).

The HSY region includes three sub-regions: two evolutionary strata whose gene order is inverted in the Y and Y^h^ sequences, compared with the X region and the orthologous region in a closely related outgroup species, and in which the X and Y sequences have diverged; and a “collinear region” with the same gene order in the X, Y, and Y^h^, and highly similar sequences in all three [12]. The border of the non-recombining fully sex-linked region was further refined based on variants differing between bacterial artificial chromosomes (BACs) made from the X and Y^h^ of a single hermaphrodite plant [12, 13]. The molecular border defined in this manner extends 277 kb into the PAR beyond the genetically defined border, suggesting that part of the collinear region still recombines at a very low rate [12]. Here, we compare sequence diversity of the different genome regions and show that the X-linked region has strikingly low diversity, even compared with closely adjacent PARs.

Recombination suppression between the papaya Y- and X-linked regions by a pericentromeric inversion in the Y chromosome is estimated to have begun seven million years ago, causing the chromosome’s pericentromeric region to become sex-linked, forming a first evolutionary stratum [12]. The Y-linked region’s larger size is largely due to repetitive element accumulation, forming four Y-specific heterochromatic knobs [14–17]; the X-linked region is also highly repetitive and shares one knob with the Y. A second stratum is estimated to have stopped recombining with the corresponding Y-linked region about 1.9 million years ago (MYA) [12]. Despite its young age in comparison with the sex chromosomes of mammals, birds or *Drosophila*, there is evidence of genetic degeneration of the papaya Y and Y^h^ [18], including the presence of pseudogenes and loss of genes [12, 19]. Finally, YY and YY^h^ genotypes abort in the embryo stage 25–50 days after pollination [20], indicating that the Y and Y^h^ have lost (or lost function of) at least one essential developmental gene in addition to carrying a gene that abolishes female functions.

We previously analyzed papaya PAR and Y-linked loci obtained by whole genome sequencing and showed that the HSY haplotype in domesticated hermaphrodites (Y^h^) is extremely similar to the MSY3 haplotype found in males in northwest Costa Rica, but not in other natural populations, and we concluded that domestication involved a hermaphrodite from this source about 4000 years ago [19]. The X in domesticated hermaphrodites should also be derived from this source population, and this study tests this. Because the X- and Y-linked regions share most genes, the system is ideal for comparing X and Y diversity and population subdivision. Here we present the first such analysis of the complete X- and Y-linked regions.

## Results

### Identification of polymorphisms and annotation

We studied the same samples of wild males and cultivated hermaphrodites (Additional file 1: Table S1) as for our previous work on the Y^h^ and Y chromosomes: 12 cultivated hermaphrodites, representing a collection of commercial papaya varieties from around the world with varied fruit quality, size, shape, color, and disease resistance and 24 wild male papaya individuals collected from three natural populations in Costa Rica [19, 21]. Additionally, we sequenced female genomes from ten cultivated varieties in order to assign variants as X- versus Y-linked and eliminate errors associated with Y reads mapping to the X region in the male and hermaphrodite samples. Our whole-genome re-sequencing (Additional file 1: Table S2) generated a total of 126 Gb of paired-end sequence reads, with an average coverage per individual of 15.6× for autosomal loci and X-linked loci in females and the expected lower coverage of the X-linked alleles (7.8×) in our male and hermaphrodite individuals. To analyze the X-linked region, the quality-filtered reads (see “Methods”) were aligned to the X region pseudomolecule [12]; for analyses of autosomal genes and the PAR, reads were aligned to the papaya draft genome [22]. We used strict parameters to avoid mapping reads from the Y-linked region to the the X region and inferred the phase of variants using reads from females, generating a set of validated X-linked haplotypes (see “Methods”). Identifying variants in the X-linked region is difficult given the highly repetitive nature of the X and its low gene density; overall, across the 3.5-Mb region, we identified a total of 12,555 SNPs not found in any Y-linked sequences, and thus probably X-specific, and 718 small insertions/deletions (X-specific indels); 193,621 SNPs and 23,825 small indels were found in the PAR sequences and 3.1 million variants were identified across the autosome. In total, 20 polymorphic sites initially assigned as X-specific were filtered from the analysis because of contaminant Y reads. These were all in a single 1-kb region containing an X-specific pseudogene (PCpX1), which may have recently transposed from the Y region, explaining the misalignment of Y reads. Most of the X-specific SNPs are intergenic (9793) and only 65 are in the 102 kb of protein coding sequences (50 non-synonymous and 15 synonymous variants; Additional file 1: Table S3); 700 are intronic, 818 are within 5 kb upstream of transcript start sites, and 1179 are within 5 kb downstream of the stop codon. One low frequency X-specific indel causes a codon deletion in a single male from a dioecious population and two low frequency X-specific indels cause nonsense mutations in three other males.

### Extremely low nucleotide diversity and evidence of a recent selective sweep in the X region

**Fig. 1:**
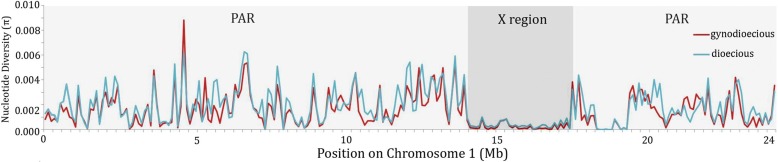
Reduced nucleotide diversity in the X region of papaya. Nucleotide diversity (π) is plotted along chromosome 1 in sliding windows of 100 kb with step size of 25 kb. Nucleotide diversity in gynodioecious papaya from domesticated samples (including X-linked regions of both hermaphrodites and females) is plotted in *red* and for wild dioecious populations (including males and females) in *blue*

**Fig. 2:**
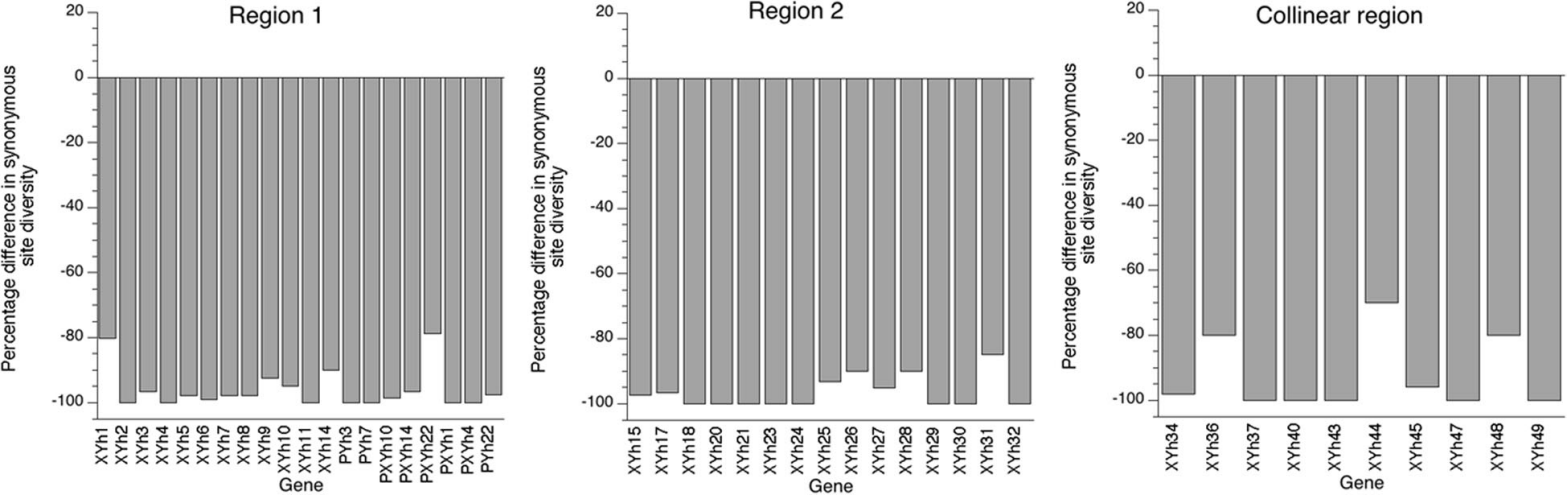
Reduced X-linked region nucleotide diversity compared to that of homologous Y-linked genes. The percentage differences in synonymous site nucleotide diversity between X and Y gene copies, relative to the diversity estimates for the Y-linked copies, are plotted for each gene with non-zero diversity in the Y-linked copy in the old stratum (*Region 1*), young stratum (*Region 2*) and the *Collinear region*. The names of the genes are shown on the *x-axes* of the plots. Four genes with one or more variants in the X-linked copy but none in the Y-linked copy were excluded from the figure, one in region 2 and three in the collinear region; the diversity among the X-linked copies of all these genes was very low (the highest value was 5 × 10^−4^ for one of the collinear region genes, about 100 times less than the average for alleles inferred to be associated with the corresponding Y-linked region)

Unexpectedly, in the natural population samples, the nucleotide diversity of X-linked genes is extraordinarily low, more than 12 times lower than predicted under neutrality, assuming that the X-linked region has an effective population size (*N*_e_) 0.75 of that for autosomal regions. The mean synonymous site nucleotide diversity (*π*_syn_) for X-linked genes (Tables 1 and 2; Additional file 1: Table S4) is 0.00017, versus 0.0018 for all autosomal genes identified; the mean estimated nucleotide diversity for all sites (*π*) is 0.00038 for the X-linked region, again much lower than values for the autosomal region or PAR (0.0017 and 0.0020, respectively, which do not differ significantly (Wilcoxon rank sum *P* = 0.12), while the difference from the X-linked region is highly significant (*P* < 1 × 10^−5^) for both regions). Figure 1 shows that the low diversity affects the entire X-linked region, ending sharply at the PAR boundaries, which makes it unlikely that a mutation rate difference is responsible (see discussion). It also cannot be explained by the fact that part of it is pericentromeric, and recombines rarely, so that the processes outlined above reduce diversity in both the X- and Y-linked regions; as explained above, the *N*_e_ value should still be three times higher for the X- than the Y-linked region, and the Y should therefore have much lower nucleotide diversity. However, the X-linked copies of almost all paired X/Y genes (in both the younger and older strata) also have much lower *π*_syn_ than the corresponding Y copies, except for a few genes in the collinear region (Fig. 2). The mean *π* for the Y is 0.0021, similar to that for the autosome. The Y value is high because Y chromosome haplotypes are strongly subdivided between populations, and the lack of recombination prevents Y-linked variants migrating between populations unless the complete haplotype migrates; the haplotype sequences can therefore diverge. Nucleotide diversity estimates in the Y-linked region within three previously identified, differentiated subpopulations (MSY1, MSY2, and MSY3) are 0.0017, 0.0010, and 0.0009, respectively, and the diversity estimates for the X-linked region in the corresponding subpopulations are statistically indistinguishable from these, also with very low values of 0.00012, 0.00011, and 0.00011, respectively.

**Table 1:** Summary statistics for population genetics tests in papaya

Chromosomal region	*π*_syn_	π	Tajima’s D	Δπ	Fst
Autosome	0.0018	0.0017			
PAR	0.002	0.002			
X-linked X	0.00017	0.00038			
X wild			−0.12	−0.76	
X cultivated			1.02	−0.63	
X-linked wild versus X cultivated					0.05
PAR wild versus PAR cultivated					0.11

**Table 2:** Summary of statistical comparisons between regions in papaya

Comparison	*P* values* for Wilcoxon signed-rank tests
X-linked π_syn_ versus autosomal π_syn_ × π_syn_ versus autosome π_syn_	1 × 10^−5^
X-linked π versus autosomal π × π versus autosome π	2.4 × 10^−6^
X-linked π versus PAR π × π versus PAR π	4.3 × 10^−6^
PAR π versus autosomal π × PAR π versus autosome π	0.14
D X_wild_ versus D X_cultivated_	1.5 × 10^−4^
Δπ X_wild_ versus Δπ X_cultivated_	1.5 × 10^−3^

*Based on Wilcoxon sign-rank tests, to take account of the different sample sizes being compared

In the cultivated hermaphrodite papayas, nucleotide diversity in the autosome is slightly lower (0.0017) than in the wild plants (Fig. 1), consistent with a loss in diversity due to a population bottleneck during domestication, though the difference is not statistically significant (Wilcoxon test *P* = 0.09). Nucleotide diversity in the X-linked region is also lower than in the wild plants (*π* for all site types = 0.0002, *π*_syn_ = 0.00013, 65% of the wild value).

**Fig. 3:**
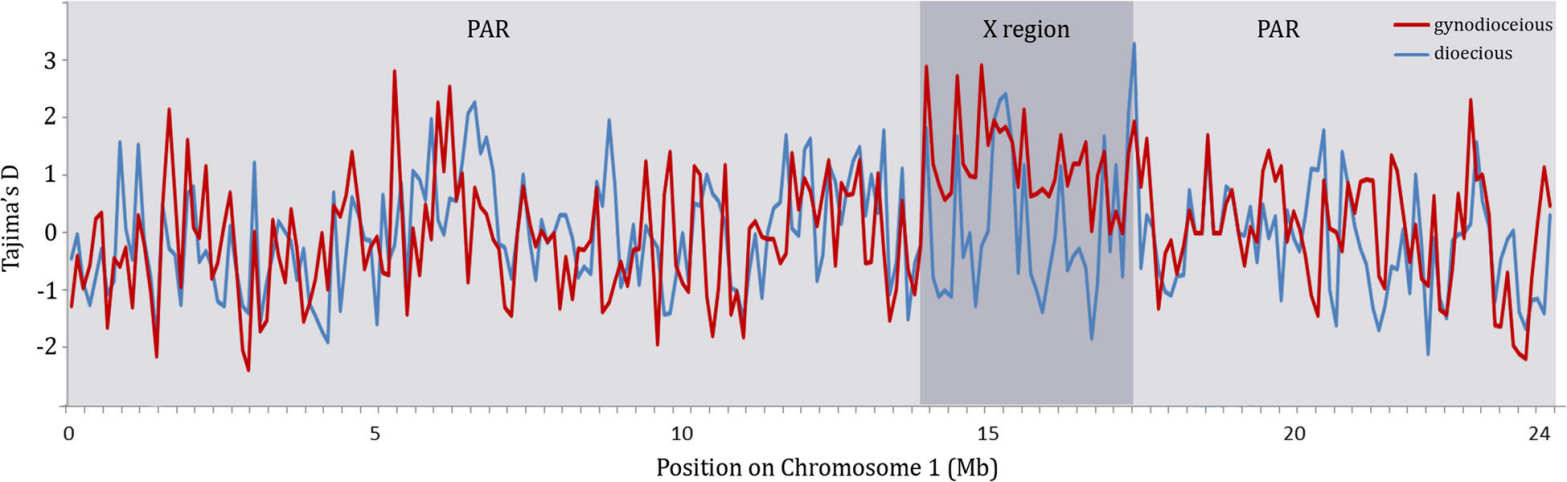
Tajima’s D across the X region and PAR of papaya. Tajima’s D values are plotted in sliding windows of 100 kb with step size of 25 kb. *Red* shows values in gynodioecious papaya and *blue* those for dioecious natural populations

A severe bottleneck that caused almost complete loss of variability should create an excess of rare variants (due to subsequent mutations not having had time to reach high frequencies), but a less severe bottleneck (or contraction of a population) would lead to loss of rare variants, while variants at high frequencies would be less likely to be lost, leaving an excess of the latter, compared with the expectation under a constant population size. An excess of rare alleles can also be caused by a selective sweep recent enough that diversity, and variant frequencies, have not yet reached the expected equilibrium values [23]. We therefore calculated Tajima’s D values, which can detect an excess or deficiency of rare variants (D values are negative in the first case above and positive in the second). Very low diversity was found for Y-linked genes in papaya hermaphrodites, compared with males, and D values were indeed much more negative, consistent with a severe bottleneck during domestication [19]. The D values for PAR sequences are close to zero in cultivated hermaphrodite papayas and only slightly more negative than in wild plants (Fig. 3), suggesting that the bottleneck during the domestication of papaya did not simply involve a single plant but specifically involved selection for a Y-linked trait, most likely the loss of the female-suppressing factor that produced hermaphroditism. In contrast, in the sample from cultivated papaya the X-linked region has more positive D values (mean D = 1.02; Fig. 3), also consistent with a moderately severe bottleneck due to sampling from a population with some diversity or to mixing between populations. This value is significantly higher than in wild papaya, whose D value is −0.12 (Wilcoxon test *P <* 0.001). The mean values of Δπ, an alternative metric to Tajima’s D that is less affected by differences in the sequence lengths analyzed [24], are −0.63 for the cultivated X and −0.76 for the wild X, a slight, but again significant, difference (Wilcoxon test *P <* 0.01), but not significantly different between the cultivated and wild PAR (−0.71 versus −0.78, respectively). This is consistent with previous findings based on four X-linked genes [25].

### Population structure in the X region and the PAR in the wild population and between wild and domesticated populations

The papaya Y-linked regions (hermaphrodite Y^h^ and male Y) show stronger population structure than that observed for the PAR sequences [19]. As explained above, the Y-linked region’s non-recombining situation makes it susceptible to genetic drift and loss of variability during population bottlenecks, especially if *N*_e_ is further reduced by hitch-hiking processes. Y sequences from wild males fall into three haplotypes and the hermaphrodite Y^h^ sequences closely resemble wild male Y haplotypes from the North Pacific region of Costa Rica [19]. If the X-linked region also lacks recombination, of this region should behave similarly. We therefore assessed the population structure for X-linked and PAR genes, using the high quality SNP and indel variants in the X-linked region and PAR described above.

**Fig. 4:**
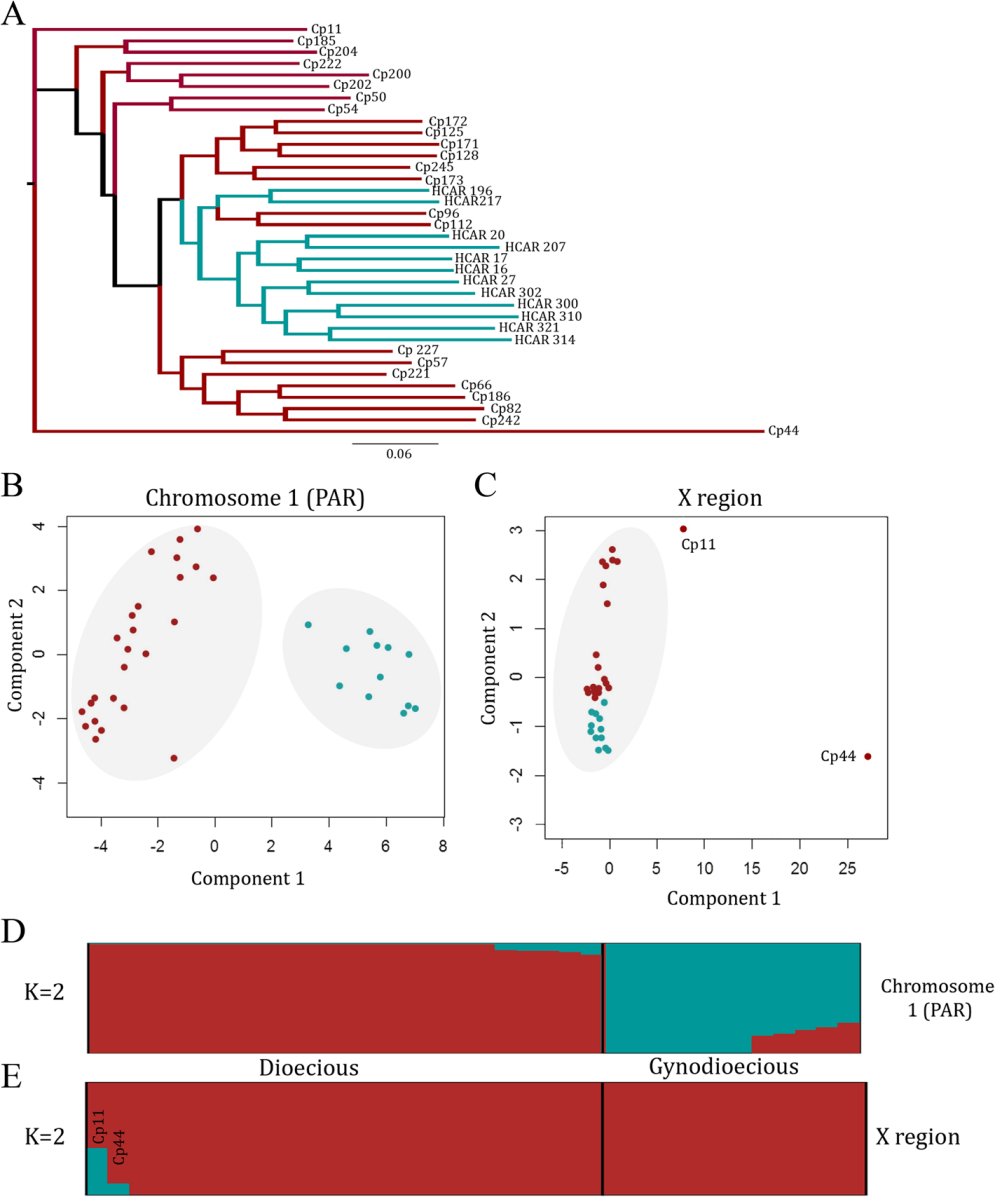
Contrasting population structure of the X region and the PAR. Gynodioecious papaya (hermaphrodites) are shown in *blue* and males from dioecious natural populations in *red*. We used 13,259 X-specific and 217,446 PAR SNPs/indels for all analyses. **a** Maximum likelihood phylogeny based on X-specific polymorphisms constructed in SNPhylo. **b** principal component analysis (PCA) based on all PAR SNPs/indels. The two distinct clusters separating male and hermaphrodite autosomes are *circled in grey*. **c** PCA based on all X-specific SNPs/indels. The single cluster containing both males and hermaphrodite Xs is *circled* and the outliers *Cp11* and *Cp44* are labeled. **d** Population structure analysis of PAR using STRUCTURE. Each accession is represented by a *vertical bar* and the length of each *colored segment* represents the contribution of each subgroup. **e** Population structure analysis of the X-linked region showing that STRUCTURE assigns the sequences from the dioecious and gynodioecious samples to a single population

Maximum likelihood phylogeny, principal component analysis (PCA), and STRUCTURE analysis of X-linked sequences all suggest a largely undivided population (although two individuals, Cp11 and Cp44, appear as outgroups in the tree based on X-linked sequences, while their PAR and Y-linked sequences do not support a separation, and two Xs, Cp96 and Cp112, from other dioecious populations fail to cluster with the other Xs; Fig. 4a, c). Gene flow clearly occurs between the wild populations. In contrast, both PCA and STRUCTURE analyses of the PAR region indicate separation between the wild and cultivated samples (Fig. 4b, d). The estimated phylogeny also groups the X sequences of cultivated hermaphrodites with X haplotypes from the dioecious subpopulations that have the MSY3 Y haplotype, as expected if the original hermaphrodite that was domesticated carried an X and a Y^h^ haplotype from such a population.

**Fig. 5:**
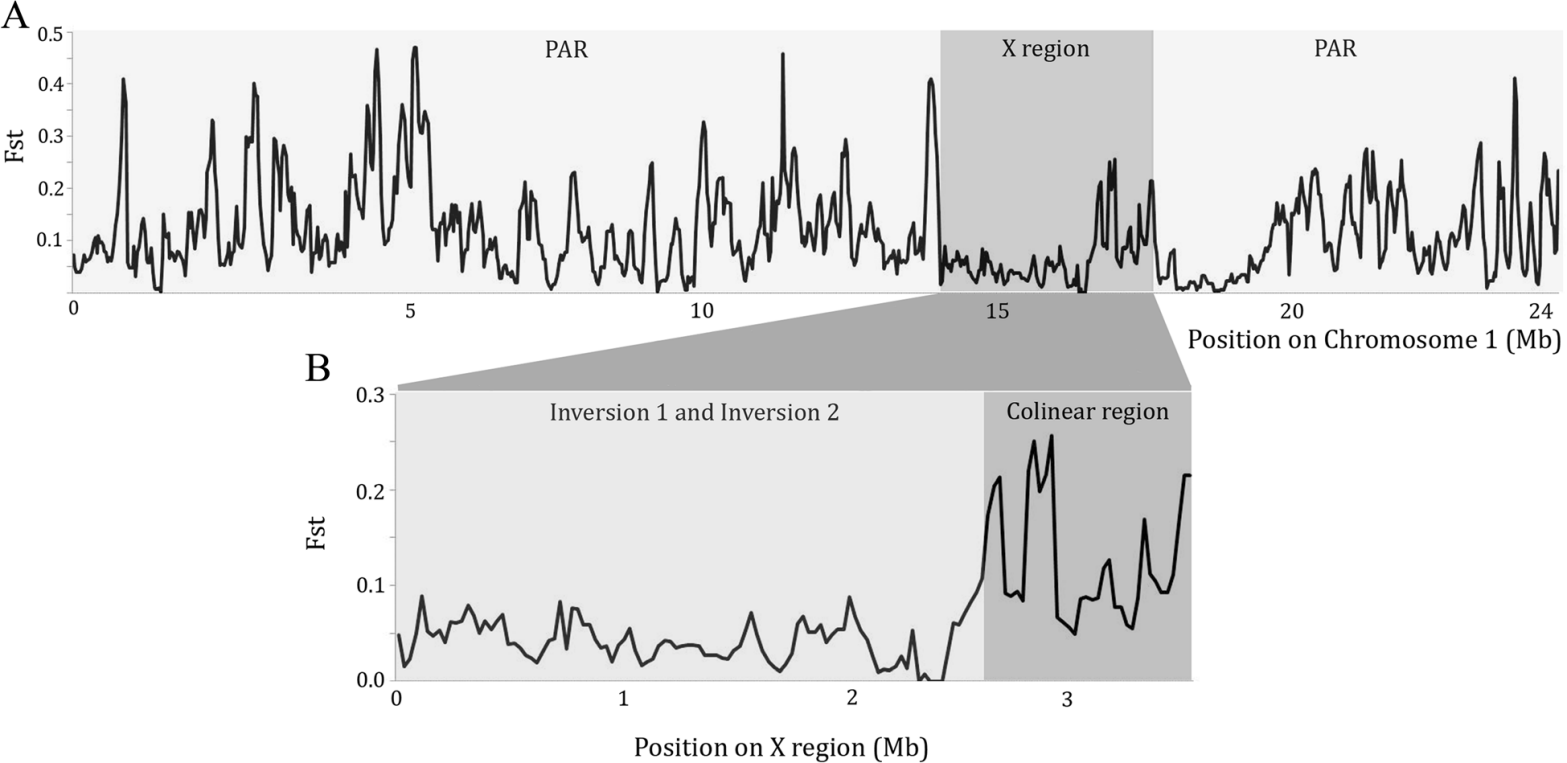
Genetic distance (*F*_*ST*_) between the dioecious and gynodioecious papaya samples using the PAR and fully X-linked regions. **a** Mean *F*_*ST*_ values between gynodioecious and dioecious papaya strains, plotted in sliding windows of 100 kb with step size of 25 kb. **b** Mean *F*_*ST*_ values for the X-specific region. The two inversion regions correspond to the two evolutionary strata (see text)

Consistent with these results, *K*_*ST*_ (a measure of population differentiation based on variance in variant frequencies between subpopulations, which is more suitable for recombining sequences than *F*_*ST*_) is only 0.05 between the X-linked sequences from the wild and domesticated populations (Fig. 5) and is 0.11 for PAR sequences, which suggests little differentiation from wild papaya following domestication as previously found [26]. The lower value for X-linked than PAR genes is surprising as their lower diversity should lead to higher *F*_*ST*_, and indeed it is higher for Y-linked than PAR genes, as expected [19]. The low *F*_*ST*_ for X-linked genes suggests some interbreeding with wild papaya following domestication, perhaps via back-crosses to wild plants, which are occasionally used in papaya breeding programs to introduce disease resistance genes, or occasional gene flow from feral hermaphrodites to wild plants, which is known to occur.

The collinear region yields a mean *F*_*ST*_ value for the wild populations similar to that for PAR genes (0.14; Fig. 5b), consistent with the evidence mentioned above that 277 kb (50%) of this collinear region at the border of the fully sex-linked region is recombining, i.e., is partially, rather than fully, sex-linked [12].

## Discussion

Extremely low sequence diversity of the entire papaya X-linked region was first suspected when a domesticated hermaphrodite and a wild dioecious papaya were found to have nearly identical X-linked sequences. Our re-sequencing of 36 genomes confirms this unexpected situation. What might have caused this? Because they recombine only in females, X-linked sequences will, on average, experience lower recombination frequencies two-thirds of those for autosomal genes. Estimates for female meiosis for five intervals in the 3.5-Mb papaya X-linked region resulted in a higher recombination rate (4.3 cM/Mb) than for 17 intervals in the PAR (1.2 cM/Mb) or the genome-wide average (2.9 cM/Mb), despite a small portion of this region being pericentromeric [27]. The high recombination rate in the papaya X-linked region in female meiosis then predicts that the rate in the population, which determines silent site diversity, is 2.88 cM/Mb, considering that X chromosomes recombine at two-thirds the rate of autosomal genes in a population (4.3 cM/Mb × 2/3), about the same as the autosomal average of 2.9 cM/Mb. The recombination rate in the X-linked region cannot, therefore, account for its low diversity. The higher recombination rate in the X-linked region is likely due to the higher DNA sequence identity of the X-linked region than that of the PAR, as indicated by the extremely low nucleotide diversity we reported here. Each pair of homologous chromosomes has one to two crossovers per meiosis and the increased frequency of recombination in the X-linked region would reduce the frequency of recombination in the PAR. This predicts nucleotide diversity values 75% of those for autosomal sequences, as explained above. However, the difference we observe is many fold larger than this. The distribution of males and females in wild papaya populations is somewhat variable, but most surveys suggest a ratio close to 1:1, with a slight excess of females. Hermaphrodites are the product of human domestication and are only found in the wild near regions with papaya cultivation. Chavez-Pesqueira et al. [28] found populations of wild papaya with 62% females, suggesting that the effective population size could be slightly higher for the X-linked region than predicted under an even sex ratio, and thus predicting a higher expected nucleotide diversity for the X, the opposite of our results, which are therefore conservative. The extremely low sequence diversity of the X-linked sequences from wild dioecious populations, together with the lack of population structure, suggests that hitch-hiking processes have reduced diversity in the populations surveyed here. Hitch-hiking includes both selective sweeps and removal of deleterious mutations [29–31], but the papaya sex-linked region is physically small and includes modest numbers of genes (50 of the 96 genes have both X- and Y-linked alleles), so that a large diversity reduction is not expected through removal of deleterious mutations and a selective sweep seems more likely, as explained below.

However, another possibility is a low mutation rate for X-linked sequences. Because X-linked regions spend a higher proportion of time in females, compared with autosomal genes, a higher mutation rate in males than females results in a lower mutation rate for the X than the autosomes [32, 33]. It is not known whether papaya has a sex difference in mutation rate, as is observed for some genes in another plant, *S. latifolia* [34]. Ideally, an Hudson–Kreitman–Aguadé (HKA) test should be done to establish whether the diversity for X-linked genes is significantly lower than expected, after taking account of mutation rate differences between different loci or genome regions [35]. Although there is a suitable outgroup species, *Vasconcellea monoica*, only five X-linked genes have been sequenced from it [36], so we are currently unable to do this test. However, this explanation can be excluded because an implausibly large mutation rate difference between PAR and X-linked genes would be required to account for the 12-fold diversity difference (or a more than 16-fold difference, taking account of the difference in *N*_e_). Mutation rate differences in [34] are detectable only in the older stratum genes, implying that, in papaya, any such difference is likely to be minor. Taken together, therefore, the low nucleotide diversity in the X-linked region suggests a strong selective sweep caused by the spread of a beneficial mutation in the region.

Our evidence further suggests that this event reduced diversity throughout the species before domestication. Our previous population structure analyses based on PAR sequences detected subdivision between wild and domesticated papaya [19]. In contrast, the X-linked sequences cluster almost all individuals into a single group, regardless of which type of population they originated from, and the very low diversity in these sequences makes discrimination between populations very difficult. The low diversity of the X-linked sequences across the entire set of all these populations implies that the selective sweep that affected the X-linked region caused spread of an X haplotype throughout a large geographic region, suggesting that strong selection was involved. It will be interesting in the future to study wild papaya populations from other regions (papaya is also found in other regions of Central America) to discover the geographic extent of this event and test whether the entire species was affected. The size of the genome region affected by a sweep depends on the recombination rate and the selection coefficient (*s*) [37] and can be roughly estimated as *d* = 0.01 *s*/*c*, where *d* is the number of bases affected and *c* is the recombination rate per base pair in Morgans. This equation was used to estimate the selection coefficient that caused the selective sweep at the *tb1* gene in maize [38]. We used it to assess whether a sweep with a plausible selection coefficient could have removed diversity across the entire 3.5-Mb X-linked region of papaya. The papaya recombination rate can be roughly estimated based on the total genome size of 372 Mb, and the estimated total genetic map length of 1069 centiMorgans (cM) [39] suggests a value of 2.9 cM/Mb. This is intermediate between estimates from maize (with a large genome size) of 0.73 cM/Mb [40] and the higher values for plants with smaller genomes such as *Arabidopsis thaliana* [41] and rice [42]. Even assuming 5 cM/Mb, a plausible *s* value of 0.05 predicts that a sweep could eliminate diversity across a 3.5-Mb region. Strong selective sweeps have also been inferred in human and great ape X chromosomes [30, 43–45], and, as expected, the nucleotide diversity is much lower for the Y-linked than X-linked genes, while the autosomal sequences have slightly higher diversity than X-linked ones. The reduction in X-linked gene diversity in papaya is much more extreme than any of these cases, and the much lower diversity than in the Y-linked genes is unprecedented in any known sex chromosome system, suggesting a recent strong selective sweep in papaya sex chromosomes evolved seven million MYA, compared with the mammalian sex chromosomes, which evolved 167 MYA. A previous study of four genes, all with both X- and Y-linked copies, found evidence for lower diversity of the former than the latter in natural populations [46]. The gene or genes causing these selective sweeps are unknown. However, the prospects for identifying the gene involved are higher in papaya because of the small number of potential candidates, 12 multi-exon X-specific genes versus nearly a thousand such genes in the human X chromosome [47]. The mutation that caused the sweep cannot be the one that is essential for embryo development, whose absence from the Y and Y^h^ chromosome leads to abortion of YY, YY^h^, and Y^h^Y^h^ genotypes [20], because abortion is due to a Y-linked loss of function mutation. The recombination suppression event that created the younger evolutionary stratum also cannot be the cause of the selective sweep, as it is probably due to inversions in the Y-linked region [12]. A recessive or partially recessive male-beneficial mutation is possible, since such mutations have a higher probability of establishing in a population if they are X-linked and the Y chromosome has no corresponding allele than if they are autosomal [48]. The papaya observations can be explained by such a mutation in one of the 12 hemizygous multi-exon genes in the papaya X-linked region homologous to the MSY and HSY, or else by a strong selective advantage acting in both sexes; in total, 34 papaya X-specific genes are absent from the Y and therefore hemizygous, but 22 of them have short single exons and their functions are unknown [12], though they are likely to be retrotransposon-mediated new genes without functions [49]. Alternatively, a partially dominant mutation that benefitted females, or both sexes, could be involved; the papaya Y-linked region also carries a functional copy of 56 genes present on the X.

## Conclusions

The extreme reduction of X-linked diversity in papaya contrasts with patterns observed in other sex systems, such as those of humans and great apes, or in the neo-X chromosome of *Drosophila miranda.* X-linked diversity is predicted to be higher than Y-linked diversity because of stronger genetic drift and hitchhiking effects as well as suppressed recombination. Our evidence further suggests that the dramatic reduction in diversity occurred prior to human domestication in contrast to the low Y^h^-linked diversity, which occurred through positive selection of hermaphroditism during early papaya cultivation. Low X-linked diversity is the product of a strong selective sweep that likely occurred in one of the 12 hemizygous multi-exon genes. Despite the separate dioecy and gynodioecy breeding systems, the X chromosomes are highly similar and clustered into a single group. This contrasts with the two subgroups (gynodioecy and dioecy) observed in the autosomes and three subgroups observed in the MSY and HSY region. The resources presented here will expedite the discovery of the sex determination genes in papaya and other genes with sex-specific benefits.

## Methods

### Sample preparation and sequencing

Wild male papaya plants were collected from ten dioecious populations around Costa Rica (Additional file 1: Table S1). Cultivated hermaphrodite (gynodioecious) papaya plants were collected from the USDA tropical plant germplasm collection in Hilo Hawaii. Fresh tissue samples from Costa Rica were dried on silica gel in the field and stored at −80 °C and fresh leaf tissue from cultivated varieties was collected from greenhouse-grown plants and stored at −80 °C. Genomic DNA was extracted from young leaf tissues using the DNAeasy Plant Mini Kit (Qiagen, Valencia, CA, USA). Paired-end DNA-seq libraries with an average insert size of 400 bp were made using the Illumina DNAseq kit according to the manufacturer’s instructions (Illumina) and sequenced on an Illumina HiSeq 2500 at 100 bp length.

### Read alignment and polymorphism identification

A total of 1.26 billion paired end reads were generated, representing an average of 15.6× coverage for autosomal and pseudo-autosomal loci and 7.8× coverage for the X-linked region in males, as expected, as they have only a single copy of X-linked genes in this region. Raw reads were filtered to remove low quality bases and trimmed for indexes using Trimmomatic (v.0.32) [50] prior to alignment. Illumina sequence adaptors were removed, leading low quality (below quality 3) and N (undetermined) base pairs were trimmed, and reads were scanned using a 4-bp sliding window and trimmed when the average quality per base dropped below 30. Clean reads were aligned to the papaya draft genome sequence [22] and the X pseudomolecule [12]. The Burrows–Wheeler Aligner [51] was used for read alignment using strict alignment parameters. The average sequence divergence between the X and Y sequences is 5–6%, but genic regions and the collinear region are less diverged (up to 3%).

To prevent the alignment of Y or autosomal reads to the X region, strict alignment and filtering parameters were used as previously reported in [19]. These criteria are briefly summarized below. We also resequenced female (XX) plants from ten cultivated accessions to verify X-specific SNP calling. Though the X and Y sequences have 5–6% sequence divergence, the two regions are largely unalignable due to the recent large scale inversions and numerous retrotransposon insertions. This divergence allowed accurate alignment of X- and Y-based reads. The last 100 kb of the collinear region, whose sequence divergence between the X and Y^h^ is <2% (based on published BAC sequences [12]), and which may recombine rather than being fully sex-linked, was excluded from our analyses of population structure and phylogeny, nucleotide diversity, *F*_*ST*_, and Tajima’s D since there are no distinguishable X and Y regions. To phase reads in the remaining fully sex linked region, strict parameters were used, including reducing the fraction of missing alignments (−n = 0.015) and high mismatch penalty (−M) with a maximum mismatch of three positions per read. The resulting list of variants was compared to a separate list of variants produced from ten female samples which are devoid of any Y-specific read mis-alignment. Any variants present at high frequency (>90%) in the male/hermaphrodite samples but absent in the female samples were classified as contaminants from Y-specific reads aligning to the X region. Only 20 sites were classified as Y contaminants and all of the sites were within a 1-kb window between 39,804 and 40,610 bp in the X region. This region corresponds to an X-specific pseudogene (PCpX1) which may have transposed from the Y region, explaining the erroneous read alignment. This suggests X–Y haplotype phasing and the low X-linked diversity are accurate.

The SAMtools package [52] was used for identifying SNPs and small indels in the X and PAR regions of chromosome 1. The raw file of unfiltered SNPs and indels was generated using mpileup under default parameters from the sorted BAM file output from bwa. Variants were called using all of the individuals concurrently, verifying the accuracy of low frequency or low coverage SNPs/indels. The unfiltered SNPs and indels include 17,324 X-linked variants and 254,312 PAR variants. Low coverage and repetitive variants were removed from the raw vcf file if they had <4 or >20–60× coverage, depending on the coverage of each individual accession. Variants with a collective root mean square (RMS) and mapping qualities (PHRED scores) <25 were removed from further analysis. Any polymorphism with more than one allele was removed as the X regions are haploid in the sequenced males/hermaphrodite plants and these variants were either repeats or Y alleles. After filtering, 12,555 SNPs and 718 indels were retained in the X-linked region and 193,621 SNPs and 23,825 indels in the PAR. SNPs in the coding region were annotated for amino acid substitutions using the papaya gene models [22] and X transcripts [12] using the program SNPeff [53].

### Population structure analyses

Maximum likelihood phylogenies were generated using a total of 217,446 high quality variants from the PAR of chromosome 1 and 13,273 variants from the X chromosome using SNPhylo [54]. SNPhylo is a highly automated package that aligns variants from a vcf file using MUSCLE and constructs a maximum likelihood phylogenetic tree using dnaml. Trees were visualized using FigTree (v.1.4; http://tree.bio.ed.ac.uk/software/figtree/). Population structure was determined using the same variants in the program STRUCTURE [55]. The methods outlined in [30] were used to infer the number of clusters (K) in the population. STRUCTURE results were plotted in distruct v1.1 [56]. The PCA was performed using PCO software.

### Population genetics analyses

*F*_*ST*_ was estimated using pair-wise comparisons of the gynodioecious lines and dioecious lines in the program SFselect (https://github.com/rronen/SFselect). Nucleotide diversity (π) and Tajima’s D were calculated in sliding windows of 100 kb with 25 kb overlap using a suite of programs in SAMtools [52]. Δπ was calculated as described in [24]. Synonymous site nucleotide diversity was calculated using aligned X genes in DnaSP [57].

## Additional file

Additional file 1: Table S1. Collection sites of wild Costa Rican papaya. **Table S2.** Summary of sequencing statistics of re-sequenced papaya genomes. **Table S3** Annotation of polymorphisms. **Table S4.** Synonymous site diversity for genes in the X-linked region. (DOCX 37 kb)
