# Supplementary material for: Extremely low nucleotide diversity in the X-linked region of papaya caused by a strong selective sweep
Source: Genome Biol. 2016 Nov 28;17:230. doi: 10.1186/s13059-016-1095-9 (PMC5125041; doi:10.1186/s13059-016-1095-9)
Supplement: Additional file 1: Table S1. — Collection sites of wild Costa Rican papaya. Table S2. Summary of sequencing statistics of re-sequenced papaya genomes. Table S3 Annotation of polymorphisms. Table S4. Synonymous site diversity for genes in the X-linked region. (DOCX 37 kb) [file 13059_2016_1095_MOESM1_ESM.docx]

**Additional file 1**

**Table S1.** Collection sites of wild Costa Rican papaya

| **Accession** | **Sex** | **GPS Coordinates** | **Operational Population** | **Region** |
| --- | --- | --- | --- | --- |
| Cp11 | M | 9° 46.538’ N, 82° 54.546’ W | 1 | Caribbean |
| Cp44 | M | 9° 49.713’ N, 82° 55.339’ W | 1 | Caribbean |
| Cp50 | M | 10° 01.641’ N, 83° 15.118’ W | 2 | Caribbean |
| Cp54 | M | 10° 02.655’ N, 83° 16.768’ W | 2 | Caribbean |
| Cp57 | M | 10° 03.886’ N, 83° 22.046’ W | 2 | Caribbean |
| Cp66 | M | 10° 04.157’ N, 84° 46.187’ W | 3 | N Pacific |
| Cp112 | M | 10° 13.565’ N, 85° 07.665’ W | 4 | N Pacific |
| Cp82 | M | 10° 27.753’ N, 85° 08.870’ W | 5 | N Pacific |
| Cp96 | M | 10° 27.810’ N, 85° 08.304’ W | 5 | N Pacific |
| Cp125 | M | 9° 55.915’ N, 84° 57.774’ W | 6 | Nicoya |
| Cp128 | M | 9° 55.314’ N, 84° 57.558’ W | 6 | Nicoya |
| Cp171 | M | 9° 53.855’ N, 84° 56.173’ W | 6 | Nicoya |
| Cp172 | M | 9° 53.867’ N, 84° 56.175’ W | 6 | Nicoya |
| Cp173 | M | 9° 53.870’ N, 84° 56.180’ W | 6 | Nicoya |
| Cp242 | M | 9° 35.267’ N, 84° 37.294’ W | 7 | Central Pacific |
| Cp245 | M | 9° 41.125’ N, 84° 39.064’ W | 7 | Central Pacific |
| Cp185 | M | 9° 24.150’ N, 84° 04.735’ W | 8 | Central Pacific |
| Cp188 | M | 9° 23.384’ N, 84° 03.696’ W | 8 | Central Pacific |
| Cp202 | M | 9° 12.835’ N, 83° 49.199’ W | 9 | S Pacific |
| Cp203 | M | 9° 12.842’ N, 83° 49.200’ W | 9 | S Pacific |
| Cp204 | M | 9° 12.838’ N, 83° 49.201’ W | 9 | S Pacific |
| Cp221 | M | 9° 00.221’ N, 83° 34.209’ W | 10 | S Pacific |
| Cp222 | M | 9° 00.221’ N, 83° 34.208’ W | 10 | S Pacific |
| Cp227 | M | 8° 59.947’ N, 83° 33.782’ W | 10 | S Pacific |
|  |  |  |  |  |

**Table S2.** Summary of sequencing statistics of re-sequenced papaya genomes.

| **Name** | **Origin** | **Cultivar** | **Sex** | **No. of Read 1** | **No. of Read 2** | **Coverage** |
| --- | --- | --- | --- | --- | --- | --- |
| Cp11 | Dioecious | NA | M | 30,596,007 | 30,596,007 | 13.3 |
| Cp44 | Dioecious | NA | M | 45,068,687 | 45,068,687 | 19.6 |
| Cp112 | Dioecious | NA | M | 37,729,972 | 37,729,972 | 16.4 |
| Cp203 | Dioecious | NA | M | 38,088,943 | 38,088,943 | 16.6 |
| Cp204 | Dioecious | NA | M | 37,218,734 | 37,218,734 | 16.2 |
| Cp54 | Dioecious | NA | M | 29,190,589 | 29,190,589 | 12.7 |
| Cp57 | Dioecious | NA | M | 25,835,569 | 25,835,569 | 11.2 |
| Cp66 | Dioecious | NA | M | 17,529,174 | 17,529,174 | 7.6 |
| Cp82 | Dioecious | NA | M | 40,331,523 | 40,331,523 | 17.5 |
| Cp96 | Dioecious | NA | M | 39,844,156 | 39,844,156 | 17.3 |
| Cp125 | Dioecious | NA | M | 52,530,869 | 52,530,869 | 22.8 |
| Cp128 | Dioecious | NA | M | 45,642,555 | 45,642,555 | 19.8 |
| Cp221 | Dioecious | NA | M | 46,838,557 | 46,838,557 | 20.4 |
| Cp172 | Dioecious | NA | M | 51,569,790 | 51,569,790 | 22.4 |
| Cp173 | Dioecious | NA | M | 25,526,766 | 25,526,766 | 11.1 |
| Cp242 | Dioecious | NA | M | 20,800,737 | 20,800,737 | 9 |
| Cp245 | Dioecious | NA | M | 46,641,984 | 46,641,984 | 20.3 |
| Cp185 | Dioecious | NA | M | 22,674,546 | 22,674,546 | 9.9 |
| Cp50 | Dioecious | NA | M | 16,498,230 | 16,498,230 | 7.2 |
| Cp188 | Dioecious | NA | M | 28,451,281 | 28,451,281 | 12.4 |
| Cp202 | Dioecious | NA | M | 31,997,441 | 31,997,441 | 13.9 |
| Cp171 | Dioecious | NA | M | 41,564,101 | 41,564,101 | 18.1 |
| Cp222 | Dioecious | NA | M | 57,093,669 | 57,093,669 | 24.8 |
| Cp227 | Dioecious | NA | M | 122,109,612 | 122,109,612 | 53.1 |
| HCAR 16 | Gynodioecious | ‘HAES 7836' | H | 30,405,103 | 30,405,103 | 11.6 |
| HCAR 17 | Gynodioecious | 'Saipan Red' | H | 47,308,928 | 47,308,928 | 18 |
| HCAR 20 | Gynodioecious | 'Khag Naun' | H | 27,625,175 | 27,625,175 | 10.5 |
| HCAR 27 | Gynodioecious | 'Kapoho' | H | 48,174,233 | 48,174,233 | 18.4 |
| HCAR 196 | Gynodioecious | 'Higgins' | H | 26,815,590 | 26,815,590 | 10.2 |
| HCAR 207 | Gynodioecious | 'Tainung No. 5' | H | 18,931,566 | 18,931,566 | 7.2 |
| HCAR 217 | Gynodioecious | 'Puerto Rico 6-65' | H | 18,322,599 | 18,322,599 | 7 |
| HCAR 302 | Gynodioecious | Line 8 | H | 16,799,494 | 16,799,494 | 6.4 |
| HCAR 309 | Gynodioecious | 'Khak Dum' | H | 13,912,543 | 13,912,543 | 5.3 |
| HCAR 310 | Gynodioecious | 'Waimanalo' | H | 15,850,446 | 15,850,446 | 6 |
| HCAR 314 | Gynodioecious | ‘KSGS’ | H | 15,531,622 | 15,531,622 | 5.9 |
| HCAR 321 | Gynodioecious | no7 323 | H | 29,987,992 | 29,987,992 | 11.4 |
| Zhonghuang | Gynodioecious | Zhonghuang' | F | 17,281,602 | 17,281,602 | 19.2 |
| Zhongbai | Gynodioecious | Zhongbai' | F | 20,152,509 | 20,152,509 | 22.4 |
| Sunset | Gynodioecious | Sunset' | F | 16,469,011 | 16,469,011 | 18.3 |
| Kamiya | Gynodioecious | Kamiya' | F | 16,253,995 | 16,253,995 | 18 |
| CR6-16 | Gynodioecious | CR6-16' | F | 17,887,058 | 17,887,058 | 19.9 |
| Sunrise | Gynodioecious | Sunrise' | F | 18,528,089 | 18,528,089 | 20.6 |
| Red Royal | Gynodioecious | Red Royal' | F | 21,268,535 | 21,268,535 | 23.6 |
| Kapoho | Gynodioecious | Kapoho' | F | 18,935,901 | 18,935,901 | 21 |
| AU9 | Gynodioecious | AU9' | F | 17,859,040 | 17,859,040 | 19.9 |
|  |  |  |  | **Average Coverage** | | **15.6** |

**Table S3.** Annotation of polymorphisms

|  | X region | Chromosome 1 (PAR) |
| --- | --- | --- |
| Total SNPs | 12,555 | 193,621 |
| Total InDels | 718 | 23,825 |
| Intergenic | 9,793 |  |
| Intronic | 700 |  |
| Upstream | 818 |  |
| Downstream | 1179 |  |
| Non-synonymous | 50 |  |
| Synonymous | 15 |  |
|  |  |  |

**Table S4.** Synonymous site diversity for genes in the X-linked region.

| **Gene Name** | **Gene length (bp in exons)** | **Synonymous site diversity** | **Region*** | **Mean for region** |
| --- | --- | --- | --- | --- |
| CpXYh1_X | 2544 | 0.000314465 | 1 | 0.000185 |
| CpXYh2_X | 1979 | 0 | 1 |  |
| CpXYh3_X | 2299 | 4.34972E-05 | 1 |  |
| CpXYh4_X | 294 | 0 | 1 |  |
| CpXYh5_X | 824 | 0.000121359 | 1 |  |
| CpXYh6_X | 3360 | 2.97619E-05 | 1 |  |
| CpXYh7_X | 2450 | 4.08163E-05 | 1 |  |
| CpXYh8_X | 3601 | 5.55401E-05 | 1 |  |
| CpXYh9_X | 1161 | 0.000258398 | 1 |  |
| CpXYh10_X | 533 | 0.000187617 | 1 |  |
| CpXYh11_X | 3126 | 0 | 1 |  |
| CpXYh12_X | 564 | 0 | 1 |  |
| CpXYh13_X | 588 | 0 | 1 |  |
| CpXYh14_X | 1449 | 0.000138026 | 1 |  |
| CpX-1 | 355 | 0.00084507 | 1 |  |
| CpX-10 | 432 | 0 | 1 |  |
| CpX-11 | 207 | 0.000483092 | 1 |  |
| CpX-12 | 183 | 0 | 1 |  |
| CpX-13 | 382 | 0 | 1 |  |
| CpX-14 | 247 | 0 | 1 |  |
| CpX-15 | 775 | 0.000129032 | 1 |  |
| CpX-16 | 246 | 0.000406504 | 1 |  |
| CpX-17 | 186 | 0 | 1 |  |
| CpX-18 | 249 | 0.000401606 | 1 |  |
| CpX-19 | 178 | 0 | 1 |  |
| CpX-20 | 249 | 0.001204819 | 1 |  |
| CpX-21 | 474 | 0.000843882 | 1 |  |
| CpX-24 | 206 | 0.000970874 | 1 |  |
| CpX-25 | 195 | 0 | 1 |  |
| CpX-26 | 387 | 0 | 1 |  |
| PYhCpXYh3_X | 600 | 0 | 1 |  |
| PYhCpXYh5_X | 261 | 0 | 1 |  |
| PYhCpXYh7_X | 1159 | 0 | 1 |  |
| PCpX-1_X | 1185 | 0.000168776 | 1 |  |
| PCpX-2_X | 379 | 0.001319261 | 1 |  |
| PCpX-3 | 153 | 0 | 1 |  |
| PCpX-4 | 352 | 0 | 1 |  |
| PXCpXYh10_X | 1779 | 5.62114E-05 | 1 |  |
| PXCpXYh14_X | 2920 | 0.000171233 | 1 |  |
| PXCpXYh22_X | 407 | 0.0004914 | 1 |  |
| PXCpXYh6_X_v3 | 1209 | 0.000165426 | 1 |  |
| PXYhCpXYh1_X | 861 | 0 | 1 |  |
| PXYhCpXYh2_X | 360 | 0 | 1 |  |
| PXYhCpXYh4_X | 906 | 0 | 1 |  |
| PYhCpXYh2 | 369 | 0 | 1 |  |
| PYhCpXYh22_X | 3250 | 3.07692E-05 | 1 |  |
| PYhCpXYh23 | 339 | 0 | 1 |  |
| PYhCpXYh27 | 252 | 0 | 1 |  |
| CpX-29 | 465 | 0.000430108 | 2 | 0.000166 |
| CpXYh15_X | 4016 | 4.98008E-05 | 2 |  |
| CpXYh16_X | 239 | 0 | 2 |  |
| CpXYh17_X | 1357 | 7.3692E-05 | 2 |  |
| CpXYh18_X | 748 | 0 | 2 |  |
| CpXYh19_X | 831 | 0.000240674 | 2 |  |
| CpXYh20_X | 6632 | 0 | 2 |  |
| CpXYh21_X | 1304 | 0 | 2 |  |
| CpXYh22_X | 243 | 0 | 2 |  |
| CpXYh23_X | 1022 | 0 | 2 |  |
| CpXYh24_X | 1800 | 0 | 2 |  |
| CpXYh25_X | 550 | 0.000363636 | 2 |  |
| CpXYh26_X | 2799 | 7.14541E-05 | 2 |  |
| CpXYh27_X | 2516 | 7.94913E-05 | 2 |  |
| CpXYh28_X | 273 | 0.0003663 | 2 |  |
| CpXYh29_X | 1869 | 0 | 2 |  |
| CpXYh30_X | 308 | 0 | 2 |  |
| CpXYh31_X | 618 | 0.000485437 | 2 |  |
| CpXYh32_X | 748 | 0 | 2 |  |
| CpXYh33_X | 515 | 0 | 2 |  |
| CpX-32 | 170 | 0 | 2 |  |
| CpX-33 | 138 | 0 | 2 |  |
| CpX-34 | 315 | 0.000634921 | 2 |  |
| CpX-6 | 422 | 0.000473934 | 2 |  |
| CpX-8 | 1114 | 0.000448833 | 2 |  |
| CpX-9 | 507 | 0.000591716 | 2 |  |
| CpXYh34_X | 1611 | 6.20732E-05 | Collinear | 0.000158 |
| CpXYh35_X | 824 | 0 | Collinear |  |
| CpXYh36_X | 1259 | 0.000158856 | Collinear |  |
| CpXYh37_X | 1406 | 0 | Collinear |  |
| CpXYh38_X | 911 | 0 | Collinear |  |
| CpXYh39_X | 1037 | 0.000289296 | Collinear |  |
| CpXYh40_X | 1925 | 0 | Collinear |  |
| CpXYh41_X | 1250 | 0.0004 | Collinear |  |
| CpXYh42_X | 1971 | 0 | Collinear |  |
| CpXYh43_X | 1318 | 0 | Collinear |  |
| CpXYh44_X | 908 | 0.000330396 | Collinear |  |
| CpXYh45_X | 1533 | 0.000130463 | Collinear |  |
| CpXYh46_X | 498 | 0 | Collinear |  |
| CpXYh47_X | 3946 | 0 | Collinear |  |
| CpXYh48_X | 176 | 0.001136364 | Collinear |  |
| CpXYh49_X | 3456 | 0 | Collinear |  |
| CpXYh50_X | 391 | 0.000511509 | Collinear |  |

*Region refers to: inversion 1 (1), inversion 2 (2) and the collinear region (collinear).
